# Exo- and Endo-1,5-α-L-Arabinanases and Prebiotic Arabino-Oligosaccharides Production

**DOI:** 10.4014/jmb.2412.12052

**Published:** 2025-01-13

**Authors:** Ye-Rin Ju, Su Been Im, Da Eun Jung, Min Jeong Son, Chan-Young Park, Min Ho Jeon, Ju Hee Hwang, Soo Jung Lee, Tae-Jip Kim

**Affiliations:** 1 Division of Animal, Horticultural and Food Sciences, Graduate School of Chungbuk National University, Cheongju 28644, Republic of Korea; 2 Department of Food Science and Biotechnology, Chungbuk National University, Cheongju 28644, Republic of Korea

**Keywords:** Endo-1,5-α-L-arabinanase (endo-ABN), exo-1,5-α-L-arabinanase (exo-ABN), structure-function relationship, arabino-oligosaccharides (AOS), enzymatic production

## Abstract

There is growing interest in pentose-based prebiotic oligosaccharides as alternatives to traditional hexose-based prebiotics. Among these, arabino-oligosaccharides (AOS), derived from the enzymatic hydrolysis of arabinan polymers, have gained significant attention. AOS can selectively stimulate the growth of beneficial gut bacteria, including *Bifidobacterium* and *Bacteroides* species, and contribute to health-benefit functions such as blood sugar control, positioning AOS as a promising synbiotic candidate. For the industrial production of AOS, the development of efficient enzymatic processes is essential, with exo- and endo-1,5-α-L-arabinanases (exo- and endo-ABNs) playing a crucial catalytic role. Most ABNs belong to the glycoside hydrolase (GH) family 43, characterized by a five-bladed β-propeller fold structure. These enzymes hydrolyze internal α-1,5-L-arabinofuranosidic linkages, producing AOS with varying degrees of polymerization. Some ABNs GH43 were known to exhibit exo-type hydrolytic modes of action, producing specific AOS products such as arabinotriose. Additionally, exo-ABNs from GH93, which feature a six-bladed β-propeller fold, exclusively release arabinobiose through their exo-type catalytic mechanism. This review represents the first comprehensive analysis of exo- and endo-ABNs, offering scientific insights into their biotechnological potential for AOS production. It systematically compares enzyme classification, structural differences, catalytic mechanisms, paving the way for innovative applications in health, food, and pharmaceutical industries.

## Introduction

Prebiotics are defined as non-digestible dietary components that reach the colon intact and selectively stimulate the cell growth and metabolism of beneficial probiotics, thereby exerting health-promoting effects on the host [1]. The ingestion of prebiotics can induce significant shifts in the composition and functionality of the colonic microbiota. Among prebiotics, non-digestible oligosaccharides with a degree of polymerization (DP) ranging from 2 to 10 represent a predominant category. These oligosaccharides exhibit stability in the gastrointestinal tract, demonstrating resistance to enzymatic hydrolysis, acidic pH, and bile salts, while specifically enhancing the growth and biological activity of health-associated intestinal microorganisms [1, 2]. Emerging evidence indicates that polyphenols also exhibit prebiotic-like properties owing to their antioxidant and anti-inflammatory effects, facilitated by their gut microbial metabolism [3]. Despite these findings, carbohydrate-based prebiotics remain a focal area of research and industry due to their broad-spectrum bioactivity, low caloric content, and versatile applicability across pharmaceuticals, functional foods, and nutraceutical formulations [4].

Prebiotic oligosaccharides can be categorized based on their chemical composition into sucrose-based fructo-oligosaccharides (FOS), lactose-based galacto-oligosaccharides (GOS), starch-based isomalto-oligosaccharides (IMOS), soy-derived oligosaccharides, chitosan-derived oligosaccharides, hemicellulose-based xylo-/arabinoxylo-/arabino-oligosaccharides (XOS, AXOS, and AOS), and so on [4]. Of these, pentose-based oligosaccharides from hemicellulosic biomass, such as XOS, AXOS, and AOS, have gained substantial interest due to their synbiotic characteristics with health-benefit and selective bifidogenic activities [5]. Given the ubiquity and structural diversity of heteroxylans in plant cell walls, extensive research has focused on the production and functional characterization of XOS and AXOS, and their prebiotic potential [5-8].

Arabinan, a homopolysaccharide predominantly extracted from sugar beet, composed of α-1,5-linked L-arabinofuranosyl residues, often substituted with α-1,2- and/or α-1,3-linked L-arabinofuranosyl branches, serves as a valuable precursor for AOS production. The enzymatic breakdown of arabinan into AOS and L-arabinose relies on the synergistic activity of exo-acting α-L-arabinofuranosidase (ABF; EC 3.2.1.55) and endo-1,5-α-L-arabinanase (ABN; EC 3.2.1.99). Exo-type ABFs are widespread across microbial taxa and are capable of selectively cleaving α-1,2-, α-1,3-, and/or α-1,5-L-arabinofuranosidic bonds from arabinan and arabinoxylan substrates [9]. On the other hand, ABNs are comparatively rare in nature and exhibit endo-acting specificity, hydrolyzing internal α-1,5-L-arabinofuranosidic linkages to generate various AOS intermediates. To date, extensive research has focused on the biochemical properties and biotechnological applications of ABFs [9-11]. However, recent genomic studies have revealed arabinan utilization gene clusters containing ABN-encoding genes in diverse microbial species [12-15]. These discoveries highlight the emerging potential of AOS as next-generation pentose-based prebiotic candidates with functional benefits for gut health modulation [16-20]. This review systematically categorizes microbial α-1,5-specific endo- and exo-arabinanases, examining the structure-function relationships governing their enzymatic mechanisms and outlining strategies for the enzymatic production of diverse prebiotic AOS.

## Prebiotic Effects of AOS

AOS are derived from the enzymatic breakdown of arabinan, a hemicellulosic polysaccharide abundantly present in plant cell walls. Arabinan exhibits significant resistance to enzymatic hydrolysis in the gastrointestinal tract, remaining largely unaffected by intestinal epithelial enzymatic activity. This inherent resistance highlights the prebiotic potential of both arabinan and its derivative AOS, enabling them to selectively support the growth of beneficial gut microbiota [21, 22]. Prebiotic effects of AOS on the growth of probiotic microbial cells are summarized (Table 1).

*In vitro* fermentation of sugar beet arabinan (SA) and AOS by the human gut microbiota indicated that AOS with various DP determine the fermentation characteristics by human gut bacteria. Different catalytic reactions were observed in bifidobacteria depending on the DP of AOS [19]. Through confirmation of growth in 24 strains of gut bacteria under given conditions, the digestive ability in the human gastrointestinal tract and prebiotic effects in the colon were established for linear arabino-oligosaccharides (LAOS) with mainly DP 2~4. LAOS has been shown to promote the growth of *Lactobacillus brevis* ATCC 14869, *Bifidobacterium longum* ATCC 15707, and *Bacteroides fragilis* ATCC 25285, increasing the population of bifidobacteria and influencing the production of short-chain fatty acids (SCFAs) [16]. Feruloylated and non-feruloylated AOS derived from sugar beet pectin were confirmed to selectively stimulate the growth of *Bifidobacterium* spp. through *in vitro* fermentation [18]. Candidate substances, such as phenylalanine, xanthine, linoleic acid, or its derivatives, confirmed to influence microbial communities as metabolites, were found to be generated more abundantly during AOS fermentation compared to FOS [17]. Microbial strains whose growth is stimulated by AOS are predominantly identified as bifidobacteria and bacteroides, with limited prebiotic effects observed in certain lactobacilli. The limited research on the prebiotic effects and health benefits of AOS, compared to L-arabinose monosaccharides and arabinan polymer, can be attributed to the absence of established mass-production technology for AOS. To address this limitation, it is crucial to discover novel ABNs with diverse hydrolytic properties and develop robust industrial-scale AOS production technologies.

## Microbial Arabinan Utilization Systems

Recent advancements in biological big data, including genomic and proteomic information, have facilitated the comprehensive analysis of microbial genomes, accelerating the identification and functional characterization of novel genes encoding ABNs with promising industrial applications. The L-arabinose/arabinan utilization gene cluster consists of genes involved in the enzymatic degradation of arabinan and AOS, as well as those related to AOS transport and L-arabinose metabolism. Typically, carbohydrate utilization gene clusters encode a variety of exo-/endo-type and intracellular/extracellular hydrolases that function synergistically in substrate breakdown. While a-1,2-/a-1,3-specific ABF genes can be found in both arabinan and arabinoxylan utilization gene clusters, a-1,5-specific ABN genes are exclusively localized within arabinan utilization clusters. Consequently, ABN-encoding genes serve as specific genetic markers for identifying and characterizing arabinan utilization gene clusters, which provides a powerful tool for exploring microbial pathways related to arabinan metabolism and facilitating biotechnological applications [15].

Previous studies investigating the structure and function of genes involved in arabinan utilization have been conducted across a diverse range of microorganisms such as *Bacillus subtilis* [25, 26], *Bacteroides* spp. [13, 27], *Hypocrea jecorina* [28], *Bifidobacterium longum* spp. [15, 29], *Geobacillus stearothermophilus* [14], *Lactobacillus crispatus* [30], and *Aspergillus niger* [31]. These studies have provided valuable insights into the genetic organization, regulatory mechanisms, and enzymatic properties of arabinan-degrading enzymes, contributing to our understanding of microbial arabinan utilization and its potential for biotechnological applications.

## Classification and Characterization of ABNs

As mentioned above, arabinan is a pectic polysaccharide with an α-1,5-L-arabinofuranosyl backbone and α-1,2 and/or α-1,3-L-arabinofuranosyl branches. Complete degradation of arabinan into L-arabinose units is achieved by the synergistic action of two types of hydrolases, exo-acting ABF (EC 3.2.1.55) and endo-ABN (EC 3.2.1.99). ABF mainly debranches α-1,2 and/or α-1,3-linked side chains, whereas ABN hydrolyzes α-1,5-linked backbone of arabinan to produce AOS. According to the CAZy (Carbohydrate-active enzymes database; http://www.cazy.org/), enzymes involved in substrate degradation are classified into distinct glycoside hydrolase (GH) families based on their catalytic mechanisms and sequence/structural homology [32]. Among these, most ABFs are distributed across GH families such as GH51, 43, and 61, while ABNs are predominantly found in GH43, characterized by a five-bladed β-propeller-shaped catalytic domain structure [33]. Almost all ABNs, except for a limited number of exo-1,5-α-L-arabinanases GH93 (exo-ABNs GH93), are known to share the structural fold of GH43. Microbial ABNs GH43 are divided into two enzyme groups, endo- and exo-ABNs, which are distinguished based on the overall structure of the catalytic cleft and the structure of the glycon site, determining their endo- or exo-action on arabinan or AOS [33-36]. The protein information and enzymatic properties of various microbial endo-ABNs GH43 known to date are compared and summarized (Table 2).

Endo-ABNs GH43 can randomly hydrolyze internal α-1,5-L-arabinofuranosidic backbone of debranched (linear) and/or branched substrates. These hydrolases demonstrate higher catalytic activity on debranched arabinan (DA) and LAOS compared to branched substrates such as sugar beet arabinan (SA) [26, 34, 38]. While the enzymatic hydrolysis efficiency on SA is relatively low, endo-ABNs GH43 can generate branched AOS (BAOS) with diverse DP and branching patterns from SA [13, 27, 42, 44]. In contrast, short-chain LAOS, predominantly with DP ranging from 2 to 4, can be produced through the endo-hydrolysis of DA or long-chain LAOS. This selective enzymatic behavior highlights the substrate specificity and product distribution of endo-ABNs, which are critical factors for optimizing the industrial production of targeted AOS with desired structural and functional properties.

Exo-1,5-α-L-arabinanases (exo-ABNs) are categorized into two distinct glycoside hydrolase families, GH43 and GH93 (Table 3). Exo-ABNs belonging to GH43 exhibit a five-bladed β-propeller architecture, sharing structural similarities with endo-ABNs GH43, particularly in the substrate-binding site conformation.

Despite these structural parallels, an ABN GH43 from *Cellvibrio japonicus* (CjArb43A) displays a unique exo-type hydrolysis pattern, selectively producing only arabinotriose from DA substrate [33, 51]. In contrast, ARN3, isolated from the bovine ruminal metagenome, demonstrates an entirely different exo-type catalytic behavior, producing only L-arabinose as the final hydrolysis product [52]. Unlike the conventional ABNs GH43, the other group of exo-ABNs GH93 possesses a six-bladed b-propeller fold structure, which primarily releases arabinobiose from DA, but hardly hydrolyzes SA [53-58]. Their modes of action are distinctly different from endo-ABNs, which typically generate a series of short-chain AOS through random internal cleavage of α-1,5-L-arabinofuranosidic bonds. These findings underscore the functional diversity among exo-ABNs, despite their structural similarity to endo-ABNs, and highlight the substrate specificity and unique product profiles dictated by subtle differences in the active site architecture and substrate interaction mechanisms.

## Structure and Catalytic Mechanism of ABNs

**Fig. 1:**
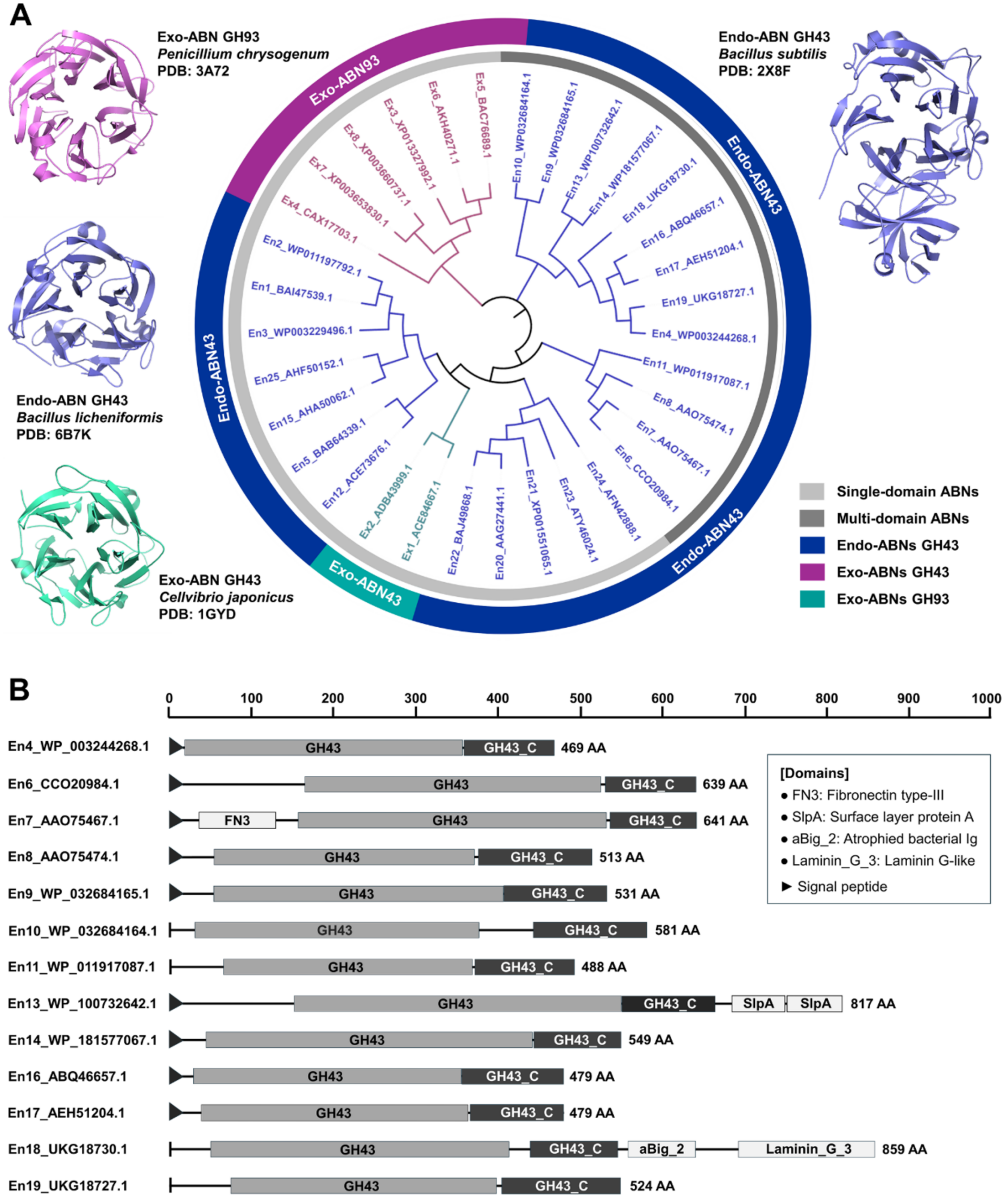
(A) Phylogenetic relationship of endo- and exo-arabinanases GH43 and GH93 and (B) Structures of multi-domain endo-arabinanases GH43. The phylogenetic tree was drawn using MEGA11 software and iTOL web server. Multi-domain structures were analyzed using InterPro and SignalP web servers. The numbering assigned to each endoand exo-ABNs corresponds to the identifiers presented in Tables 2 and 3. The black triangle indicates a signal peptide for protein secretion.

The three-dimensional structures of endo- and exo-ABNs GH43 predominantly feature a catalytic domain with a five-bladed β-propeller fold. This structural motif facilitates the enzymatic breakdown of arabinan and AOS with high specificity and efficiency. However, a subset of exo-ABNs GH93 possesses a six-bladed β-propeller fold in their catalytic domain, distinguishing them structurally and functionally from ABNs GH43. To elucidate the structural differences and similarities between these ABNs, comparative analyses of their primary and tertiary structures were performed and comprehensively illustrated (Fig. 1).

Phylogenetic analysis of various ABNs based on amino acid sequence identity revealed a clear evolutionary distinction between exo-ABNs GH93 and endo-ABNs GH43 (Fig. 1A). This result highlights significant differences in amino acid sequences and catalytic actions between exo-ABNs GH93 and ABNs GH43, reflecting their functional and structural divergence. In contrast, two exo-ABNs GH43 exhibit higher sequence similarity with single-domain endo-ABNs GH43 than with exo-ABNs GH93. This closer evolutionary relationship suggests a shared structural and functional ancestry between exo- and endo-ABNs GH43, despite their distinct catalytic modes of action. Furthermore, some endo-ABNs GH43 possess multi-domain architectures, comprising an additional C-terminal domain alongside the catalytic domain for GH43 hydrolase. Beyond these core domains, certain ABNs include optional domains such as fibronectin type-III (FN3) domain, surface layer protein A (SlpA) domain, atrophied bacterial Ig (aBig) domain, or laminin-G domain, respectively (Fig. 1B).

Comparative structural analysis provides critical insights into the molecular mechanisms underlying substrate recognition and catalytic specificity of ABNs GH43 and GH93. These contribute significantly to their potential biotechnological applications, particularly in the targeted production of AOS. The first resolved three-dimensional structure of an ABN GH43 with a five-bladed β-propeller fold was reported for CjArb43A from *Cellvibrio japonicus* [33]. This enzyme contains three essential catalytic acidic residues within its active site and hydrolyzes arabinan and AOS into arabinotriose via an inverting catalytic mechanism. Three-dimensional structures of endo-ABNs GH43 in complex with AOS substrates have been elucidated from *Geobacillus thermodenitrificans* (PDB: 6A8I with arabinohexaose) [59], *G. stearothermophilus* (3D61 with arabinobiose, 3D5Z with arabinotriose) [34], and *Bacillus subtilis* (2X8S with arabinotriose) [35]. Additionally, the structures of a thermostable endo-ABN GH43 from *Thermotoga petrophila* RKU-1 (4KC8) and ARN2 GH43 discovered from the bovine rumen metagenome (4KCA) were also reported [60].

The crystal structures of an intracellular, monomeric, and single-domain endo-ABN GH43 (GsAbnB; 315 amino acids) from *Geobacillus stearothermophilus* T-6, in complex with various AOS, revealed the mechanism of substrate-binding and catalytic action of typical endo-ABNs GH43.

The first acidic residue (E201) of GsAbnB acts as a general acid, protonating the leaving aglycon and making it a better leaving group. The second acidic residue (D27) acts as a general base, activating a water molecule for a single-displacement attack on the anomeric carbon, resulting in the inversion of the anomeric configuration. The third residue (D147) probably maintains the correct alignment of the general acid residue with the substrate, modulates the pK_a_ of the general acid residue, and stabilizes transition states such as the oxocarbenium ion through interactions with the 2-OH of the glycon [33, 34, 61-63]. The structures of GsAbnB have been comparatively analyzed in complex with arabinobiose (PDB: 3D61), arabinotriose (3D5Z), and arabinopentaose (6F1G). Isothermal titration calorimetry (ITC) analysis of catalytic mutants of GsAbnB interacting with various AOS revealed that the active site comprises at least five subsites (-2, -1, +1, +2, and +3) capable of accommodating arabinopentaose [34]. The catalytic triad residues (D27, D147, and E201) are precisely positioned with appropriate spatial distances and orientations to hydrolyze an α-L-arabinofuranosidic linkage between L-arabinose units at subsites -1 and +1. Arabinobiose is challenging to hydrolysis because it primarily binds to the +1 and +2 subsites of the enzyme, preventing proper positioning within the catalytic cleft. As a result, GsAbnB exhibits extremely low hydrolytic activity on arabinobiose, preventing its decomposition into L-arabinose monomers. In the case of arabinotriose, it binds weakly to the subsites -1, +1, and +2 and is slowly decomposed into L-arabinose and arabinobiose by GsAbnB. Arabinopentaose interacts with all five subsites (-2, -1, +1, +2, and +3) within the active site. This extended binding interaction enhances substrate affinity and supports efficient catalytic activity, making arabinopentaose and longer substrates such as DA the preferred substrates for GsAbnB.

**Fig. 2:**
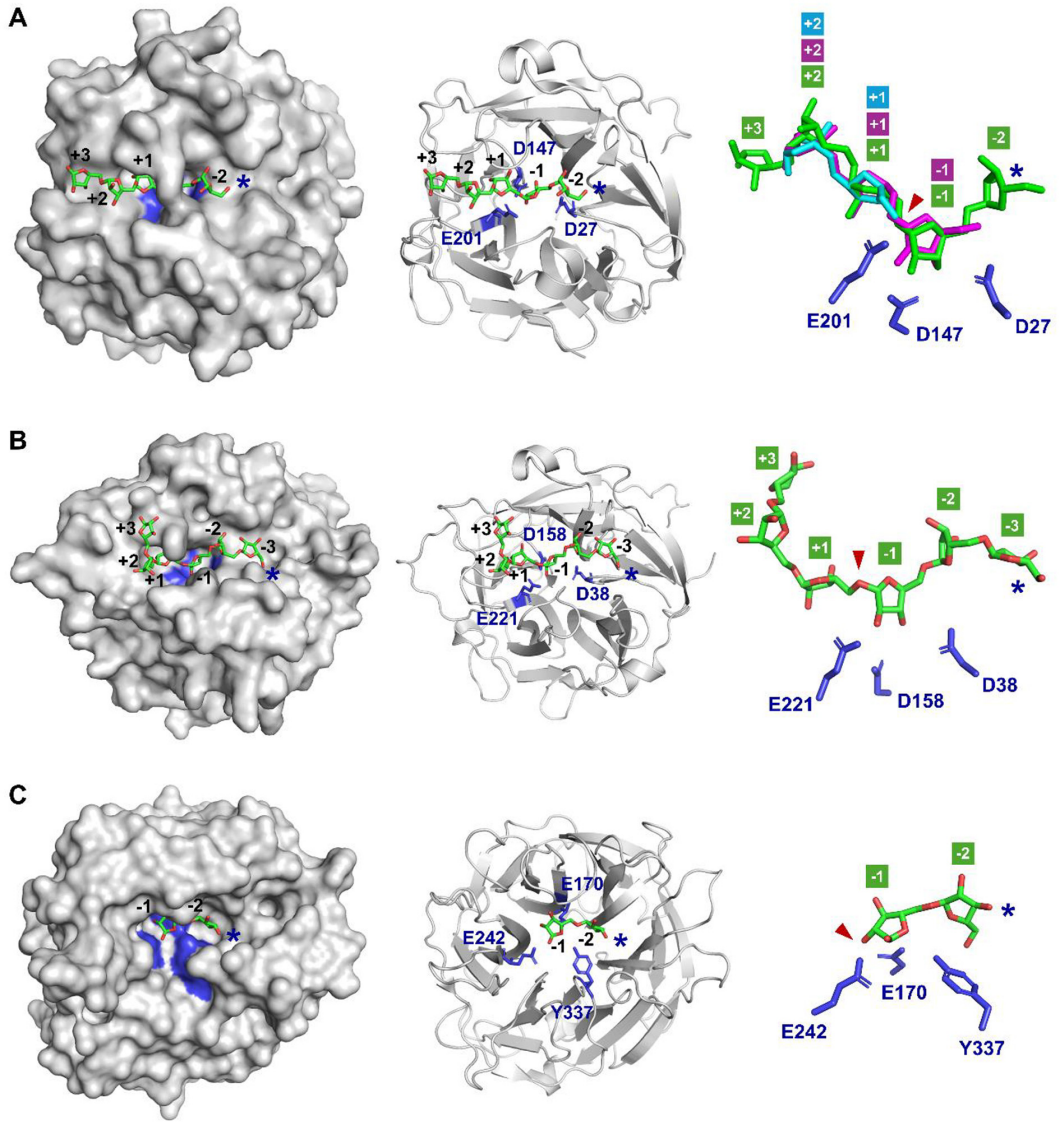
Three-dimensional structures of microbial exo- and endo-1,5-α-L-arabinanases (ABNs) GH43 and GH93 in complex with substrates. Surface, cartoon, and closed-up structures are represented for (**A**) endo-ABN GH43 from *Geobacillus stearothermophilus* (PDB: 3CU9, catalytic residues of D27, D147, and E201 in blue) in complex with arabinobiose (3D61 in cyan), arabinotriose (3D5Z in magenta), and arabinopentaose (6F1G in green), (**B**) exo-ABN GH43 from *Cellvibrio japonicus* with arabinohexaose (PDB: 1GYD and 1GYE, catalytic residues of D38, D158, and E221 in blue), and (**C**) exo-ABN GH93 from *Penicillium chrysogenum* with arabinobiose (PDB: 3A72, catalytic residues of E170, E242, and Y337 in blue). The substrate-binding site for each enzyme is illustrated with designated subsite numbers. The substrate cleavage sites and non-reducing ends of substrates are marked with arrowheads and asterisks, respectively. Each structure is visualized using PyMOL molecular graphics tool.

Exo-ABNs are enzymes known for their exo-acting hydrolytic activity, and they exhibit different modes of action compared to endo-ABNs and exo-ABFs. For instance, an exo-ABN GH43 from *Cellvibrio japonicus* (CjArb43A, PDB: 1GYD) releases only arabinotriose from the non-reducing end of arabinan [33, 51] (Fig. 2B). Although CjArb43A shares a typical five-bladed β-propeller structure with common endo-ABNs GH43, it differs in the loop sequence connecting the second and the third blades. This loop sequence consists of 13 amino acids, in contrast to 4-6 amino acid residues found in the short loop of typical endo-ABNs GH43. However, the loop structure in CjArb43A has been confirmed not to influence its exo-type modes of action. Mutagenesis studies revealed that the amino acid residues responsible for steric restrictions at subsite -3 play a crucial role in its hydrolytic modes of action, leading to a transition from exo-acting to endo-acting activity [64]. CjArb43A is structurally very similar to the typical endo-ABNs GH43 but has a high substrate affinity at subsite -3, so that the long-chain substrates can bind in a specific orientation and position, exhibiting the exo-acting hydrolysis characteristics. In contrast, ARN3 (PDB: 4KCB), an exo-ABN GH43 discovered from the rumen metagenome, was known to have a loop structure (28 amino acids) affecting its exo-acting activity [60]. ARN3 catalyzes the exo-acting degradation of linear arabinan from the reducing end to produce L-arabinose units [52]. When the loop sequence of ARN3 was replaced with that of endo-ABN (SRGEEP, 5 amino acids), the mutant ARN3 showed increased catalytic efficiency, enhanced substrate affinity, and a shift to an endo-acting mode of action, respectively [60]. While most exo-acting ABNs GH43 are known to act on the non-reducing end, only a few exo-hydrolases were confirmed to act on the reducing end.

Some exo-ABNs have a six-bladed β-propeller fold structure, classifying them under the GH93 family. The Abnx from *Penicillium chrysogenum* 31B is an exo-ABN belonging to the GH93 family and hydrolyzes DA from the non-reducing end to release only arabinobiose as a product [55]. The structure of Abnx (PDB ID: 3A72) was shown in complex with arabinobiose (Fig. 2C). It is difficult to understand the exact substrate binding and hydrolysis mechanism of exo-ABNs GH93 because a few complex structures with substrates have been elucidated to date. Based on the structure in complex with arabinobiose, the location of subsites -1 and -2 can be predicted, which explains the structural reason why Abnx can exclusively generate arabinobiose as a product, not L-arabinose. The structure of Arb93A from *Fusarium graminearum* (PDB ID: 2W5N) is another example of exo-ABN GH93, and its modes of action involve the non-reducing end of linear arabinan [54].

## Enzymatic Production of AOS Using ABNs

**Fig. 3:**
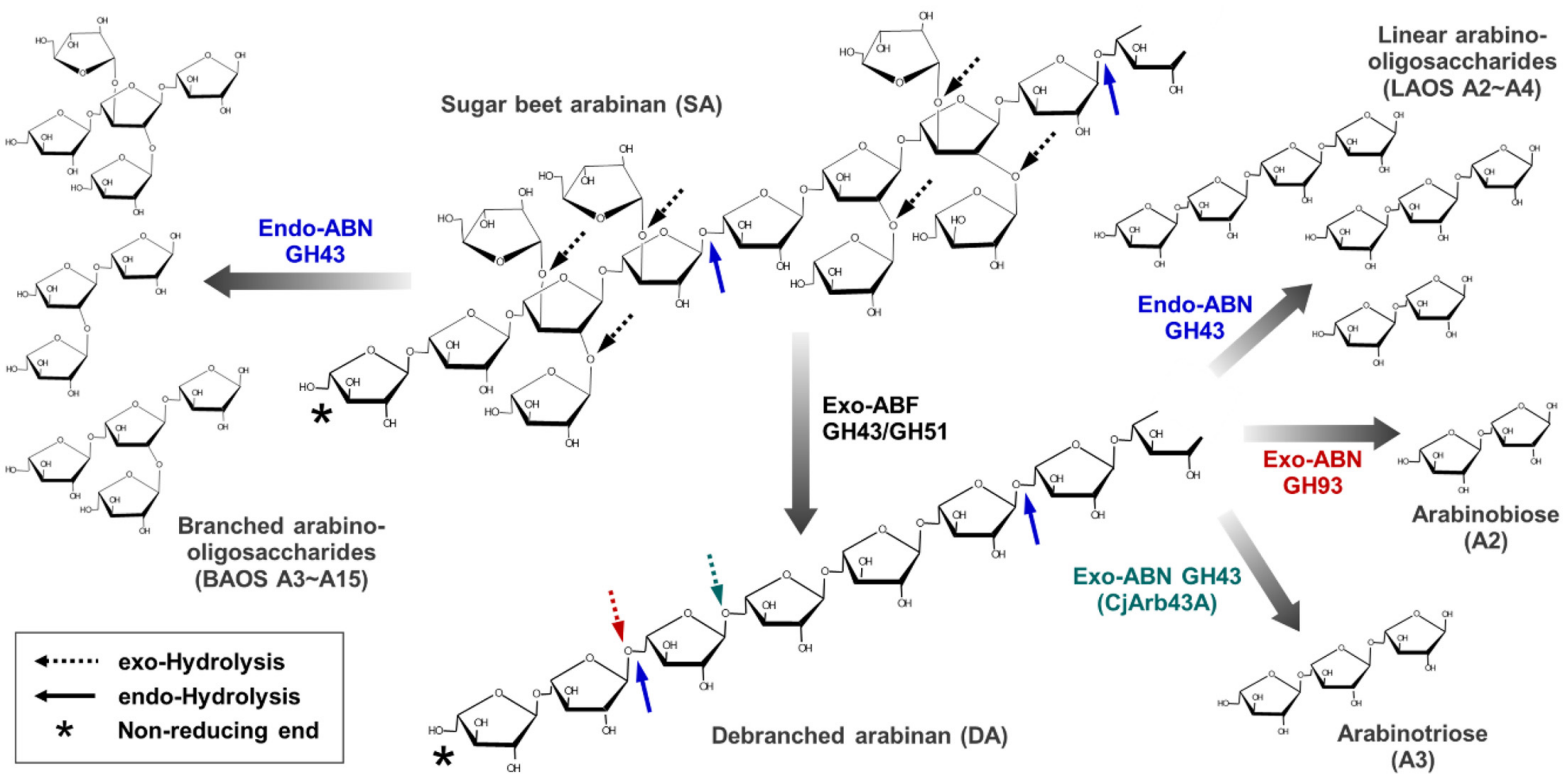
Scheme for enzymatic production of prebiotic arabino-oligosaccharides using exo- and endo-1,5-α-Larabinanases (ABNs) GH43 and GH93. CjArb43A, exo-ABN GH43 from *Cellvibrio japonicus*; exo-ABF, α-Larabinofuranosidase with debranching activity.

Since arabinan is a homopolymer composed exclusively of L-arabinose, early research primarily focused on achieving high-yield production of L-arabinose through the synergistic action of endo-ABN and exo-ABF [65, 66]. However, the complete decomposition of arabinan into L-arabinose is possible not only by enzymes but also by acid treatment. Accordingly, the research focus has shifted towards the production of AOS using endo-ABNs, as AOS exhibit prebiotic properties and functional benefits beyond L-arabinose. This transition reflects an emerging industrial interest in value-added bioproducts, where ABNs play a pivotal role in controlling the DP and structural profiles of AOS. As described above, microbial ABNs found in nature have been reported to exhibit diverse structures, substrate specificities, and hydrolysis mechanisms. In this study, it is suggested that the appropriate utilization of exo- and endo-type ABNs can enable the production of AOS with a wide range of DP and diverse chemical structures (Fig. 3).

For BAOS production, specific endo-ABNs GH43 with hydrolytic activity toward SA should be employed. While most endo-ABNs GH43 exhibit higher activity on DA, some enzymes have been reported to demonstrate significant hydrolytic activity toward SA as well. When SA is treated with these specialized endo-ABNs GH43, BAOS with varying degrees of branches and diverse branched structures can be produced through the hydrolysis of α-1,5-L-arabinofuranosidic linkages within SA. For example, BAOS were produced through the enzymatic treatment of three arabinohydrolases, Abn1 (endo-ABN), Abn2 (exo-ABN), and Abn4 (exo-ABF), derived from *Chrysosporium lucknowense* [58]. The resulting BAOS were classified into two distinct types, α-1,3-single-substituted and α-1,2-/α-1,3-double-substituted BAOS. The chemical structures of eight different BAOS were elucidated through detailed structural analysis [67]. LAOS can be produced using endo- or exo-ABNs with various hydrolytic properties, using DA as the substrate. However, the production of DA as a starting substrate is a prerequisite, which necessitates the use of ABFs capable of efficiently debranching the α-1,2- and α-1,3-linked side chains from SA. These ABFs selectively hydrolyze side-chain substitutions, enabling the conversion of branched arabinan (SA) into a linear form (DA) suitable for subsequent enzymatic hydrolysis by ABNs [68]. Once DA is generated from SA through ABF treatment, it can serve as a precursor to produce various AOS. When DA is treated with general endo-ABNs GH43, a mixture of short-chain LAOS with a DP ranging from 2 to 4 can be produced. The composition and distribution of LAOS in the mixture may vary depending on the specific hydrolytic characteristics of the ABN employed. The desired AOS fractions can be isolated and purified using column chromatography, filtration, and/or crystallization techniques. The purified AOS fractions are then utilized for further studies and functional analyses to explore their prebiotic potential and biological activities [17, 19, 20].

When exo-ABNs GH43 are employed, AOS with a specific DP can be produced, depending on the hydrolytic characteristics of the enzyme used. This selective production of single AOS species offers a significant advantage in achieving economic feasibility during the industrial downstream processes, as it simplifies successive purification and fractionation steps. For example, when exo-type CjArb43A GH43 is applied to DA, the main product is exclusively arabinotriose, depending on its unique hydrolytic modes of action. In contrast, most exo-type ABNs GH93 can predominantly produce arabinobiose, offering a targeted and efficient AOS production process. The enzyme-specific hydrolysis patterns enable precise control over AOS production, facilitating their application in functional foods, pharmaceuticals, and nutraceutical industries where consistency and structural specificity are critical.

## Future Prospectives

The demand for next-generation prebiotic carbohydrate materials as alternatives to widely used existing prebiotics is steadily increasing. Pentose-based oligosaccharides derived from hemicellulose, such as AOS, are gaining significant attention due to their potential health benefits and selective prebiotic properties. To produce structurally and functionally diverse AOS, the development of novel enzymes with unique hydrolytic properties is essential. In this review, we have presented the prebiotic effects of AOS, the classification of various ABNs available for AOS production, enzymatic properties, their structure-function relationships, and the foundation for utilizing enzymes in the efficient production of AOS. Recently, global interest in the relationship between dietary intake and gut microbiome diversity has surged, leading to an increase in research on functional synbiotics, which involves the strategic pairing of prebiotics and probiotics [69-75]. This research field aims to explore how specific combinations of prebiotic carbohydrates and probiotic strains can synergistically enhance gut health, modulate microbial composition, and improve host well-being. This requires an integrated approach, including (1) discovery and characterization of enzyme resources with novel catalytic properties, (2) development of industrial-scale mass production and purification techniques for enzymes, (3) enhancement of enzyme properties through protein engineering, (4) establishment of an efficient mass production process for prebiotic materials via enzyme treatment, (5) elucidation of the functional effects of synbiotics using advanced multi-omics (*e.g.*, genomics, transcriptomics, proteomics, metabolomics, and so on) analyses. Integrating these processes enables a comprehensive understanding of enzyme-substrate interactions, microbial metabolic pathways, and prebiotic utilization mechanisms, for the efficient design and production of next-generation synbiotics with enhanced functional properties and commercial availability. Microbial exo- and endo-ABNs are indispensable biocatalysts for the enzymatic production of prebiotic AOS, offering significant potential for applications across the health, food, pharmaceutical, and agricultural sectors. Ongoing advancements in enzyme technology will be essential for unlocking the full commercial and biotechnological potential of prebiotic AOS, paving the way for innovative applications in gut health and functional nutrition.

## Figures and Tables

**Table 1:** Prebiotic effects of arabino-oligosaccharides (AOS) on microbial cell growth.

Prebiotic AOS candidates	Growth-stimulated target microorganisms	Ref.
Linear AOS (DP 2~10)	*Bifidobacterium longum* ATCC 15707, *B. breve* ATCC 15700, *B. adolescentis* ATCC 15703, *Bacteroides vulgatus* ATCC 8482	[23]
Arabinan-hydrolyzed AOS fractions	*Bifidobacterium*, *Bacteroides* spp.	[19]
Acid-hydrolyzed AOS (DP 1~11)	*Bifidobacterium*, *Bacteroides*/*Prevotella* spp.	[24]
Linear and branched AOS (DP 2~10)	*Bifidobacterium*, *Lactobacillus* spp. (from patients with ulcerative colitis)	[20]
Feruloylated and non-feruloylated AOS	*Bifidobacterium* spp.	[18]
High (>1 kDa) and low M.W. AOS	*Bifidobacterium* spp.	[17]
Linear AOS (Mainly DP 2~4)	*Bifidobacterium longum* ATCC 15707, *Lactobacillus brevis* ATCC 14869, *Bacteroides fragilis* ATCC 25285	[16]
Branched AOS, debranched AOS	*Bacteroides cellulosilyticus*, *B. dorei*, *B. eggerthii*, *B. finegoldii*, *B. fragilis*, *B. intestinalis*, *B. massiliensis*, *B. oleiciplenus*, *B. ovatus*, *B. stercoris*, *B. thetaiotaomicron*, *B. vulgatus*, *B. xylanisolvens*	[13]

**Table 2:** Characteristics of microbial endo-1,5-α-L-arabinanases Glycoside Hydrolase (GH) 43 family.

No.	ABNs	Microbial origins	GenBank ID	AA^a^	Substrates^b^	Products^c^	Ref.
Prokaryotic endo-ABNs GH43							
1	Endo-1,5-α-L-arabinanase	*Bacillus licheniformis*	BAI47539.1	291*	DA	AOS	[37]
2	BLABNase	*Bacillus licheniformis*	WP_011197792.1	291*	DA≥LA	AOS (2-4)	[38]
3	AbnA	*Bacillus subtilis*	WP_003229496.1	291*	LA>SA	AOS	[26]
4	Abn2	*Bacillus subtilis*	WP_003244268.1	443*	LA≥SA>>P	AOS	[25]
5	ABN-TS	*Bacillus thermodenitrificans*	BAB64339.1	313	DA≥SA	AOS (2)	[39]
6	AbnA	*Bacteroides* sp. (Termite gut)	CCO20984.1	618*	SA>DA	AOS (4-5)	[27]
7	BT0360	*Bacteroides thetaiotaomicron*	AAO75467.1	622*	SA>>DA	AOS	[13]
8	BT0367	*Bacteroides thetaiotaomicron*	AAO75474.1	488*	DA>>SA	AOS	[13]
9	BflsABN43A	*Bifidobacterium longum* subsp. *suis*	WP_032684165.1	505*	DA>>SA	AOS (2-4)	[15]
10	BflsABN43B	*Bifidobacterium longum* subsp. *suis*	WP_032684164.1	581	DA	AOS (2)	[15]
11	Endo-1,5-α-L-arabinanase	*Caldicellulosituptor Saccharolyticus*	WP_011917087.1	488	DA>>SA	AOS	[40]
12	AbnB	*Geobacillus stearothermophilus*	ACE73676.1	315	DA	AOS (2-3)	[34]
13	AbnA	*Lactobacillus crispatus*	WP_100732642.1	773*	LA>SA	AOS	[30]
14	AbnB	*Lactobacillus crispatus*	WP_181577067.1	518*	LA	AOS	[30]
15	AbnZ1	*Paenibacillus polymyxa*	AHA50062.1	288*	LA>DA>SA	AOS (2-3)	[41]
16	AbnA	*Thermotoga petrophila*	ABQ46657.1	452*	SA>DA	AOS (2-3)	[42]
17	Tth Abn	*Thermotoga thermarum*	AEH51204.1	447*	LA≥DA>SA	AOS (2-3)	[43]
18	MC72GH43-2	*Xylanivirga thermophila* (Biogas reactor metagenome)	UKG18730.1	859	DA>SA	AOS (2-3)	[44]
19	MC68GH43-1	*Xylanivirga thermophila*	UKG18727.1	524	SA>DA	AOS (2-3)	[44]
Eukaryotic endo-ABNs GH43							
20	ARA1	*Aspergillus aculeatus*	AAG27441.1	302*	DA>SA	AOS (2-3)	[45]
21	BcAra1	*Botrytis cinerea*	XP_001551065.1	290*	DA≥LA	AOS	[46]
22	AbnS1	*Penicillium Chrysogenum*	BAJ49868.1	293*	DA	AOS (2-3)	[47]
23	ABN1	*Penicillium purpurogenum*	ATY46024.1	309*	DA>>SA	AOS	[48]
24	PcARA	*Phanerochaete chrysosporium*	AFN42888.1	293*	DA	AOS (2-3)	[49]
25	RmArase	*Rhizomucor miehei*	AHF50152.1	288*	DA>SA	AOS (2)	[50]

^a^Amino acid residues (AA) constituting each enzyme is presented with an asterisk if the predicted signal peptide sequence was excluded.

^b^Abbreviations: DA, debranched arabinan; LA, linear arabinan; SA, sugar beet arabinan; P, pectin; AOS, arabino-oligosaccharides.

^c^Endo-ABNs can produce linear and/or branched AOS from DA and SA, respectively, and the degrees of polymerization (DP) of resulting major linear AOS products were shown in parentheses.

**Table 3:** Characteristics of exo-1,5-α-L-arabinanases Glycoside Hydrolase (GH) 43 and 93 families.

No.	ABNs	Microbial origins	GenBank ID	AA^a^	Substrates^b^	Products	Ref.
exo-ABNs GH43							
1	Arb43A	*Cellvibrio japonicus* (*Pseudomonas cellulosa*)	ACE84667.1	316*	CMA≥LA, AOS	A3	[51]
2	ARN3	Bovine ruminal metagenome	ADB43999.1	327*	CMLA≥DA≥LA, AOS	A1	[52]
exo-ABNs GH93							
3	ReAbn93	*Rasamsonia emersonii*	XP_013327992.1	362*	A4≥A3≥LA	A2	[53]
4	Arb93A	*Fusarium graminearum*	CAX17703.1	365*	DA>>SA, AOS	A2	[54]
5	Abnx	*Penicillium chrysogenum*	BAC76689.1	354*	DA>>SAG≥SA, AOS	A2	[55]
6	Arap2	*Talaromyces purpureogenus* (*Penicillium purpurogenum*)	AKH40271.1	363*	DA>>BX≥RA≥SA, AOS	A2	[56]
7	Abn93T	*Thermothielavioides terrestris*	XP_003653830.1	364*	DA, AOS	A2	[57]
8	Abn2	*Chrysosporium lucknowense*	XP_003660737.1	361*	LA>SA, AOS	A2	[58]

^a^Amino acid residues (AA) constituting each enzyme is presented with an asterisk if the predicted signal peptide sequence was excluded.

^b^Abbreviations: CMA, carboxymethyl arabinan; LA, linear arabinan; CMLA, carboxymethyl linear arabinan; DA, debranched arabinan; SA, sugar beet arabinan; BX, birchwood xylan; RA, rye arabinoxylan; SAG, soybean arabinogalactan; AOS, arabinooligosaccharides; A1, L-arabinose; A2, arabinobiose; A3, arabinotriose; A4, arabinotetraose.

## References

[1] Gibson GR, Roberfroid MB (1995). Dietary modulation of the human colonic microbiota: introducing the concept of prebiotics. J. Nutr..

[2] Lomax AR, Calder PC (2008). Prebiotics, immune function, infection and inflammation: a review of the evidence. Br. J. Nutr..

[3] Plamada D, Vodnar DC (2021). Polyphenols-gut microbiota interrelationship: a transition to a new generation of prebiotics. Nutrients.

[4] Rajagopalan G, Krishnan C, Rathinam NK, Sani R (2019). Next Generation Biomanufacturing Technologies.

[5] Leschonski KP, Mortensen MS, Hansen LBS, Krogh K, Kabel MA, Laursen MF (2024). Structure-dependent stimulation of gut bacteria by arabinoxylo-oligosaccharides (AXOS): a review. Gut Microbes.

[6] Schmitz E, Leontakianakou S, Norlander S, Nordberg Karlsson E, Adlercreutz P (2022). Lignocellulose degradation for the bioeconomy: the potential of enzyme synergies between xylanases, ferulic acid esterase and laccase for the production of arabinoxylooligosaccharides. Bioresour. Technol..

[7] Huang C, Yu Y, Li Z, Yan B, Pei W, Wu H (2022). The preparation technology and application of xylo-oligosaccharide as prebiotics in different fields: a review. Front. Nutr..

[8] Santibáñez L, Henríquez C, Corro-Tejeda R, Bernal S, Armijo B, Salazar O (2021). Xylooligosaccharides from lignocellulosic biomass: a comprehensive review. Carbohydr. Polym..

[9] Numan MT, Bhosle NB (2006). α-L-arabinofuranosidases: the potential applications in biotechnology. J. Ind. Microbiol. Biotechnol..

[10] Poria V, Saini JK, Singh S, Nain L, Kuhad RC (2020). Arabinofuranosidases: characteristics, microbial production, and potential in waste valorization and industrial applications. Bioresour. Technol..

[11] Long L, Lin Q, Wang J, Ding S (2024). Microbial α-L-arabinofuranosidases: diversity, properties, and biotechnological applications. World J. Microbiol. Biotechnol..

[12] Arzamasov AA, van Sinderen D, Rodionov DA (2018). Comparative genomics reveals the regulatory complexity of bifidobacterial arabinose and arabino-oligosaccharide utilization. Front. Microbiol..

[13] Luis AS, Briggs J, Zhang X, Farnell B, Ndeh D, Labourel A (2018). Dietary pectic glycans are degraded by coordinated enzyme pathways in human colonic *Bacteroides*. Nat. Microbiol..

[14] Shulami S, Raz-Pasteur A, Tabachnikov O, Gilead-Gropper S, Shner I, Shoham Y (2011). The L-arabinan utilization system of *Geobacillus stearothermophilus*. J. Bacteriol..

[15] Kang Y, Choi CY, Kang J, Ju YR, Kim HB, Han NS (2024). Functional characterization of endo- and exo-hydrolase genes in arabinan degradation gene cluster of *Bifidobacterium longum* subsp. *suis*. Int. J. Mol. Sci..

[16] Moon JS, Shin SY, Choi HS, Joo W, Cho SK, Li L (2015). *In vitro* digestion and fermentation properties of linear sugar-beet arabinan and its oligosaccharides. Carbohydr. Polym..

[17] Sulek K, Vigsnæs LK, Schmidt LR, Holck J, Frandsen HL, Smedsgaard J (2014). A combined metabolomic and phylogenetic study reveals putatively prebiotic effects of high molecular weight arabino-oligosaccharides when assessed by *in vitro* fermentation in bacterial communities derived from humans. Anaerobe.

[18] Holck J, Lorentzen A, Vigsnæs LK, Licht TR, Mikkelsen JD, Meyer AS (2011). Feruloylated and nonferuloylated arabinooligosaccharides from sugar beet pectin selectively stimulate the growth of *Bifidobacterium* spp. in human fecal in vitro fermentations. J. Agric. Food Chem..

[19] Al-Tamimi MA, Palframan RJ, Cooper JM, Gibson GR, Rastall RA (2006). *In vitro* fermentation of sugar beet arabinan and arabinooligosaccharides by the human gut microflora. J. Appl. Microbiol..

[20] Vigsnæs LK, Holck J, Meyer AS, Licht TR (2011). *In vitro* fermentation of sugar beet arabino-oligosaccharides by fecal microbiota obtained from patients with ulcerative colitis to selectively stimulate the growth of *Bifidobacterium* spp. and *Lactobacillus* spp. Appl. Environ. Microbiol..

[21] Hotchkiss AT, Olano-Martin E, Grace WE, Gibson GR, Rastall RA, Eggleston G, Côté GL (2003). Oligosaccharides in Food and Agriculture.

[22] Yoo HD, Kim D, Paek SH (2012). Plant cell wall polysaccharides as potential resources for the development of novel prebiotics. Biomol. Ther..

[23] Van Laere K, Hartemink R, Bosveld M, Schols H, Voragen A (2000). Fermentation of plant cell wall derived polysaccharides and their corresponding oligosaccharides by intestinal bacteria. J. Agric. Food Chem..

[24] Onumpai C, Kolida S, Bonnin E, Rastall RA (2011). Microbial utilization and selectivity of pectin fractions with various structures. Appl. Environ. Microbiol..

[25] Inácio JM, de Sá-Nogueira I (2008). Characterization of *abn2* (*yxiA*), encoding a *Bacillus subtilis* GH43 arabinanase, Abn2, and its role in arabino-polysaccharide degradation. J. Bacteriol..

[26] Leal TF, de Sá-Nogueira I (2004). Purification, characterization and functional analysis of an endo-arabinanase (AbnA) from *Bacillus subtilis*. FEMS Microbiol. Lett..

[27] Arnal G, Bastien G, Monties N, Abot A, Anton Leberre V, Bozonnet S (2015). Investigating the function of an arabinan utilization locus isolated from a termite gut community. Appl. Environ. Microbiol..

[28] Akel E, Metz B, Seiboth B, Kubicek CP (2009). Molecular regulation of arabinan and L-arabinose metabolism in *Hypocrea jecorina* (*Trichoderma reesei*). Eukaryot. Cell..

[29] Komeno M, Hayamizu H, Fujita K, Ashida H (2019). Two novel α-L-arabinofuranosidases from *Bifidobacterium longum* subsp. *longum* belonging to glycoside hydrolase family 43 cooperatively degrade arabinan. Appl. Environ. Microbiol..

[30] Li Q, Gänzle MG (2020). Characterization of two extracellular arabinanases in *Lactobacillus crispatus*. Appl. Microbiol. Biotechnol..

[31] Seiboth B, Metz B (2011). Fungal arabinan and L-arabinose metabolism. Appl. Microbiol. Biotechnol..

[32] Drula E, Garron ML, Dogan S, Lombard V, Henrissat B, Terrapon N (2022). The carbohydrate-active enzyme database: functions and literature. Nucleic Acids Res..

[33] Nurizzo D, Turkenburg JP, Charnock SJ, Roberts SM, Dodson EJ, McKie VA (2002). *Cellvibrio japonicus* α-L-arabinanase 43A has a novel five-blade β-propeller fold. Nat. Struct. Biol..

[34] Alhassid A, Ben-David A, Tabachnikov O, Libster D, Naveh E, Zolotnitsky G (2009). Crystal structure of an inverting GH 43 1,5-α-L-arabinanase from *Geobacillus stearothermophilus* complexed with its substrate. Biochem. J..

[35] de Sanctis D, Inácio JM, Lindley PF, de Sá‐Nogueira I, Bento I (2010). New evidence for the role of calcium in the glycosidase reaction of GH43 arabinanases. FEBS J..

[36] Farro EGS, Leite AET, Silva IA, Filgueiras JG, de Azevedo ER, Polikarpov I (2018). GH43 *endo*-arabinanase from *Bacillus licheniformis*: structure, activity and unexpected synergistic effect on cellulose enzymatic hydrolysis. Int. J. Biol. Macromol..

[37] Seo ES, Lim YR, Kim YS, Park CS, Oh DK (2010). Characterization of a recombinant endo-1,5-α-L-arabinanase from the isolated bacterium *Bacillus licheniformis*. Biotechnol. Bioproc. Eng..

[38] Park JM, Jang MU, Kang JH, Kim MJ, Lee SW, Song YB (2012). Detailed modes of action and biochemical characterization of endo-arabinanase from *Bacillus licheniformis* DSM13. J. Microbiol..

[39] Takao M, Akiyama K, Sakai T (2002). Purification and characterization of thermostable endo-1,5-α-L-arabinase from a strain of *Bacillus thermodenitrificans*. Appl. Environ. Microbiol..

[40] Hong MR, Park CS, Oh DK (2009). Characterization of a thermostable endo-1,5-α-L-arabinanase from *Caldicellulorsiruptor saccharolyticus*. Biotechnol. Lett..

[41] Wang S, Yang Y, Yang R, Zhang J, Chen M, Matsukawa S (2014). Cloning and characterization of a cold-adapted endo-1,5-α-Larabinanase from *Paenibacillus polymyxa* and rational design for acidic applicability. J. Agric. Food Chem..

[42] Squina FM, Santos CR, Ribeiro DA, Cota J, de Oliveira RR, Ruller R (2010). Substrate cleavage pattern, biophysical characterization and low-resolution structure of a novel hyperthermostable arabinanase from *Thermotoga petrophila*. Biochem. Biophys. Res. Commun..

[43] Shi H, Ding H, Huang Y, Wang L, Zhang Y, Li X (2014). Expression and characterization of a GH43 endo-arabinanase from *Thermotoga thermarum*. BMC Biotechnol..

[44] Liu Y, Angelov A, Feiler W, Baudrexl M, Zverlov V, Liebl W (2022). Arabinan saccharification by biogas reactor metagenomederived arabinosyl hydrolases. Biotechnol. Biofuels Bioprod..

[45] Skjøt M, Kauppinen S, Kofod LV, Fuglsang C, Pauly M, Dalbøge H (2001). Functional cloning of an endo-arabinanase from *Aspergillus aculeatus* and its heterologous expression in *A. oryzae* and tobacco. Mol. Genet. Genomics.

[46] Nafisi M, Stranne M, Zhang L, van Kan JA, Sakuragi Y (2014). The endo-arabinanase BcAra1 is a novel host-specific virulence factor of the necrotic fungal phytopathogen *Botrytis cinerea*. Mol. Plant Microbe Interact..

[47] Sakamoto T, Inui M, Yasui K, Tokuda S, Akiyoshi M, Kobori Y (2012). Biochemical characterization and gene expression of two endo-arabinanases from *Penicillium chrysogenum* 31B. Appl. Microbiol. Biotechnol..

[48] Vilches F, Ravanal MC, Bravo-Moraga F, Gonzalez-Nilo D, Eyzaguirre J (2018). *Penicillium purpurogenum* produces a novel endo-1,5-arabinanase, active on debranched arabinan, short arabinooligosaccharides and on the artificial substrate p-nitrophenyl arabinofuranoside. Carbohydr. Res..

[49] Huy ND, Thiyagarajan S, Choi YE, Kim DH, Park SM (2013). Cloning and characterization of a thermostable endo-arabinanase from *Phanerochaete chrysosporium* and its synergistic action with endo-xylanase. Bioprocess Biosyst. Eng..

[50] Chen Z, Liu Y, Yan Q, Yang S, Jiang Z (2015). Biochemical characterization of a novel endo-1,5-α-L-arabinanase from *Rhizomucor miehei*. J. Agric. Food Chem..

[51] McKie VA, Black GW, Millward-Sadler SJ, Hazlewood GP, Laurie JI, Gilbert HJ (1997). Arabinanase A from *Pseudomonas fluorescens* subsp. *cellulosa* exhibits both an endo-and an exo-mode of action. Biochem. J..

[52] Wong DW, Chan VJ, Batt SB (2008). Cloning and characterization of a novel exo-α-1, 5-L-arabinanase gene and the enzyme. Appl. Microbiol. Biotechnol..

[53] An J, Xu W, Meng X, Chen G, Zhang W, Liu W (2022). Biochemical characterization of a thermophilic exo-arabinanase from the filamentous fungus *Rasamsonia emersonii*. J. Biosci. Bioeng..

[54] Carapito R, Imberty A, Jeltsch JM, Byrns SC, Tam PH, Lowary TL (2009). Molecular basis of arabinobio-hydrolase activity in phytopathogenic fungi: crystal structure and catalytic mechanism of *Fusarium graminearum* GH93 exo-α-L-arabinanase. J. Biol. Chem..

[55] Sakamoto T, Thibault JF (2001). Exo-arabinanase of *Penicillium chrysogenum* able to release arabinobiose from α-1,5-L-arabinan. Appl. Environ. Microbiol..

[56] Mardones W, Callegari E, Eyzaguirre J (2015). Heterologous expression of a *Penicillium purpurogenum* exo-arabinanase in *Pichia pastoris* and its biochemical characterization. Fungal Biol..

[57] Velasco J, Oliva B, Gonçalves AL, Lima AS, Ferreira G, França BA (2020). Functional characterization of a novel thermophilic exo-arabinanase from *Thermothielavioides terrestris*. Appl. Microbiol. Biotechnol..

[58] Kühnel S, Hinz S, Pouvreau L, Wery J, Schols H, Gruppen H (2010). *Chrysosporium lucknowense* arabinohydrolases effectively degrade sugar beet arabinan. Bioresour. Technol..

[59] Yamaguchi A, Sogabe Y, Fukuoka S, Sakai T, Tada T (2018). Structures of endo-1,5-α-L-arabinanase mutants from *Bacillus thermodenitrificans* TS-3 in complex with arabino-oligosaccharides. Acta Crystallogr. F. Struct. Biol. Commun..

[60] Santos CR, Polo CC, Costa MC, Nascimento AF, Meza AN, Cota J (2014). Mechanistic strategies for catalysis adopted by evolutionary distinct family 43 arabinanases. J. Biol. Chem..

[61] Pons T, Naumoff DG, Martínez-Fleites C, Hernández L (2004). Three acidic residues are at the active site of a β-propeller architecture in glycoside hydrolase families 32, 43, 62, and 68. Proteins.

[62] Jitonnom J, Hannongbua S (2018). Theoretical study of the arabinan hydrolysis by an inverting GH43 arabinanase. Mol. Simul..

[63] Meelua W, Wanjai T, Thinkumrob N, Oláh J, Mujika JI, Ketudat-Cairns JR (2022). Active site dynamics and catalytic mechanism in arabinan hydrolysis catalyzed by GH43 endo-arabinanase from QM/MM molecular dynamics simulation and potential energy surface. J. Biomol. Struct. Dyn..

[64] Proctor MR, Taylor EJ, Nurizzo D, Turkenburg JP, Lloyd RM, Vardakou M (2005). Tailored catalysts for plant cell-wall degradation: redesigning the exo/endo preference of *Cellvibrio japonicus* arabinanase 43A. Proc. Nat. Acad. Sci. USA.

[65] Lim YR, Yeom SJ, Kim YS, Oh DK (2011). Synergistic production of L-arabinose from arabinan by the combined use of thermostable endo- and exo-arabinanases from *Caldicellulosiruptor saccharolyticus*. Bioresour. Technol..

[66] Park JM, Jang MU, Oh GW, Lee EH, Kang JH, Song YB (2015). Synergistic action modes of arabinan degradation by *exo*- and *endo*-arabinosyl hydrolases. J. Microbiol. Biotechnol..

[67] Westphal Y, Kühnel S, de Waard P, Hinz SW, Schols HA, Voragen AG (2010). Branched arabino-oligosaccharides isolated from sugar beet arabinan. Carbohydr. Res..

[68] Oh GW, Kang Y, Choi CY, Kang SY, Kang JH, Lee ML (2019). Detailed mode of action of arabinan-debranching α-Larabinofuranosidase GH51 from *Bacillus velezensis*. J. Microbiol. Biotechnol..

[69] Jang HJ, Lee NK, Paik HD (2024). A narrative review on the advance of probiotics to metabiotics. J. Microbiol. Biotechnol..

[70] Ngoc APT, Zahoor A, Kim DG, Yang SH (2024). Using synbiotics as a therapy to protect mental health in Alzheimer's disease. J. Microbiol. Biotechnol..

[71] Chang D, Gupta VK, Hur B, Cobo-López S, Cunningham KY, Han NS (2024). Gut microbiome wellness index 2 enhances health status prediction from gut microbiome taxonomic profiles. Nat. Commun..

[72] Qu J, Meng F, Wang Z, Xu W (2024). Unlocking cardioprotective potential of gut microbiome: exploring therapeutic strategies. J. Microbiol. Biotechnol..

[73] Carlino N, Blanco-Míguez A, Punčochář M, Mengoni C, Pinto F, Tatti A (2024). Unexplored microbial diversity from 2,500 food metagenomes and links with the human microbiome. Cell.

[74] Xu Y, Wu X, Li Y, Liu X, Fang L, Jiang Z (2024). Probiotics and the role of dietary substrates in maintaining the gut health: use of live microbes and their products for anticancer effects against colorectal cancer. J. Microbiol. Biotechnol..

[75] Jang YJ, Min B, Lim JH, Kim BY (2023). In vitro evaluation of probiotic properties of two novel probiotic mixtures, consti-biome and sensi-biome. J. Microbiol. Biotechnol..

